# Quantitative NMR-Based Lipoprotein Analysis Identifies Elevated HDL-4 and Triglycerides in the Serum of Alzheimer’s Disease Patients

**DOI:** 10.3390/ijms232012472

**Published:** 2022-10-18

**Authors:** Georgy Berezhnoy, Christoph Laske, Christoph Trautwein

**Affiliations:** 1 Laboratory for Metabolomics and Systems Medicine, Werner Siemens Imaging Center, Department of Preclinical Imaging and Radiopharmacy, University Hospital Tübingen, 72076 Tübingen, Germany; 2 Section for Dementia Research, Hertie Institute for Clinical Brain Research, Department of Psychiatry and Psychotherapy, University Hospital Tübingen, 72076 Tübingen, Germany; 3 German Center for Neurodegenerative Diseases (DZNE), 72076 Tübingen, Germany

**Keywords:** ^1^H-NMR, serum, lipoproteins, liquor, beta-amyloid, tau, plaques, metabolism, neurodegeneration, metabolomics, Alzheimer’s disease

## Abstract

Alzheimer’s disease (AD) is the most common form of dementia in the elderly and has been associated with changes in lipoprotein metabolism. We performed quantitative lipoprotein analysis in a local cohort of cognitively impaired elderly and control subjects using standardized nuclear magnetic resonance (NMR) spectroscopy. A commercially available quantitative NMR-based assay covering 112 lipoprotein main and subtype variables was used to investigate blood serum samples from a moderate cohort size of 161 persons (71 female, 90 male), including measures of quality control. Additionally, clinical metadata and cerebrospinal fluid AD biomarkers were collected and used for analysis. High-density lipoprotein (HDL) HDL-4 subfraction levels were mostly high in female individuals with mild cognitive impairment (MCI), followed by AD. Low-density lipoprotein (LDL) LDL-2 cholesterol was slightly elevated in male AD patients. HDL-2 apolipoprotein Apo-A1, HDL-2 phospholipids, and HDL-3 triglycerides were highly abundant in AD and MCI women compared to men. When considering clinical biomarkers (Aβ, tau), very low-density lipoprotein (VLDL) VLDL-1 and intermediate-density lipoprotein (IDL) triglycerides were substantially higher in AD compared to MCI. In addition, triglyceride levels correlated positively with dementia. Different lipoprotein serum patterns were identified for AD, MCI, and control subjects. Interestingly, HDL-4 and LDL-2 cholesterol parameters revealed strong gender-specific changes in the context of AD-driven dementia. As gender-based comparisons were based on smaller sub-groups with a low n-number, several statistical findings did not meet the significance threshold for multiple comparisons testing. Still, our finding suggests that serum HDL-4 parameters and various triglycerides correlate positively with AD pathology which could be a read-out of extended lipids traveling through the blood-brain barrier, supporting amyloid plaque formation processes. Thereof, we see herein a proof of concept that this quantitative NMR-based lipoprotein assay can generate important and highly interesting data for refined AD diagnosis and patient stratification, especially when larger cohorts are available.

## 1. Introduction

Alzheimer’s disease (AD) is the most common form of dementia in the elderly worldwide [1]. AD is characterized by an accumulation of amyloid plaques and neurofibrillary tangles in the brain, provoking a significant loss of neurons and synapses, followed by severe brain atrophy in later stages [2].

Lipid metabolism in the brain is linked to brain activities that may impede cognitive ability [3]. Indeed, the lipid profiles in AD and cardiovascular diseases are closely related [4,5]. Furthermore, memory functions have been shown to correlate with lipid parameters in blood [6,7]. Thus, the precise analysis of lipids in blood seems to be a promising approach to AD research and diagnostics [8].

To date, beta-amyloid (Aβ) and tau proteins in cerebrospinal fluid (CSF) are established diagnostic biomarkers for AD [9]. Unlike CSF collection, which is associated with certain risks, lipid composition analysis based on blood samples offers an easier way to investigate patients during hospital check-ups. This is especially relevant due to the widespread availability of serum and the option of frequent sampling. Moreover, the literature suggests a set of lipid parameters detected in blood that could serve as AD biomarkers.

High-density lipoprotein (HDL) elevation is an essential biomarker of AD and may be beneficial in discriminating AD patients from healthy subjects [10,11]. Furthermore, low-density lipoprotein (LDL) cholesterol was shown to be an AD-like state predictor [12,13].

Aside from routine clinical laboratory tests, advanced analytical testing may provide a wider output for discovering lipid metabolism changes in patient blood samples. For example, several lipid species identified via mass spectrometry exhibited characteristic changes in AD dementia patients and individuals with mild cognitive impairment (MCI) [14,15,16].

Different from mass spectrometry, the use of nuclear magnetic resonance (NMR) spectroscopy provides nondestructive sample preparation and high batch reproducibility [17]. Additionally, NMR spectroscopy has the possibility of straightforward and simultaneous blood metabolite and lipid identification and quantification [18]. Indeed, NMR-based analysis has been used for AD research and diagnostics of disease progression in the context of lipoproteins [19,20,21,22].

Based on the currently reported advances in targeted lipoprotein analysis by the use of NMR spectroscopy, we proposed that an in-depth lipoprotein analysis including various subclasses could elucidate individual patients’ phenotypes and develop novel diagnostic targets for personalized precision medicine. Our approach is focused on applying an in vitro diagnostics research (IVDr) standard operation procedure (SOP) to serum aliquots with quantitative profiling of proton (^1^H) NMR spectra. This standardized approach guarantees high reliability and reproducibility [23].

The quantitative NMR-based lipoprotein data generated herein were used to elucidate AD-specific changes in metabolism and to correlate them with clinical parameters (Aβ, tau, apolipoprotein ApoE4 allele status). Additionally, using an IVDr and SOP-derived dataset will help to construct a quantitative lipoprotein knowledge base of different diseases (e.g., cardiovascular disease and diabetes) and put them into context with alterations of lipid metabolism under dementia and aging processes.

## 2. Results

### 2.1. Cohort Description

A total of 182 serum samples were initially received for NMR lipoprotein analysis. For these samples, group labeling, clinical metadata, and demographical parameters were provided. Notably, the full clinical metadata, such as Aβ1-42 and tau markers (CSF h-Tau and CSF p-Tau), were not available for all patients in the entire cohort. Three clinical groups were investigated within this study: control (Con) subjects and mild cognitive impairment (MCI) and AD patients.

The clinical data showed a general differentiation by the significant elevation of tau protein structures in both the MCI and AD groups. Amyloid peptides Aβ1-42 were significantly lower only in the CSF subcohort (Table 1).

### 2.2. NMR-Based Lipoprotein Variables from Serum

The annotation and quantification of serum spectra were provided automatically by a server-based service from Bruker BioSpin GmbH (Ettlingen, Germany). Thus, a total of 112 lipoprotein parameters (via Bruker IVDr Lipoprotein Subclass Analysis B.I. LISA™ (Bruker BioSpin GmbH, Ettlingen, Germany), analysis package; Supplementary Materials Table S1) were identified and quantified in all samples.

### 2.3. Qualitative Control (QC) of NMR Spectra Filters Out Nonsuited Datasets

The qualitative control analysis was performed automatically and was provided alongside the main lipoprotein data (B.I. LISA™) based on characteristic features of the NMR raw data using the analysis package Bruker IVDr BioBank QC B.I.BioBankQC™ (Bruker BioSpin GmbH, Ettlingen, Germany).

**Figure A1:**
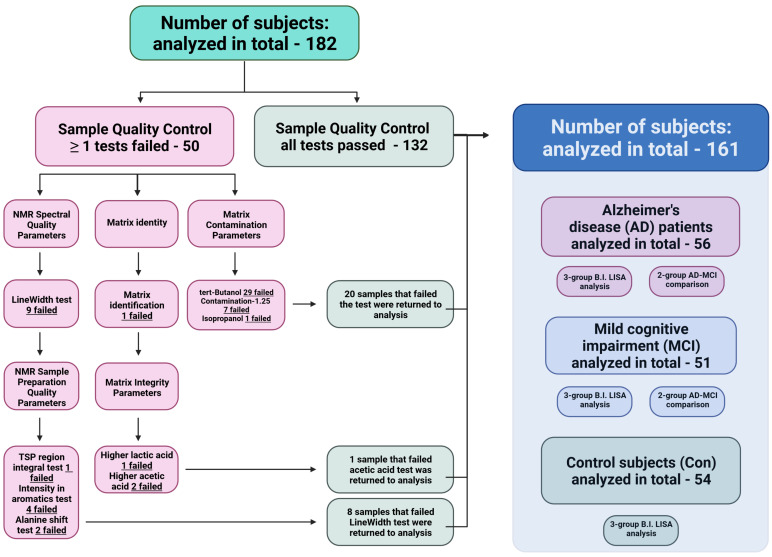
Tree diagram of QC with the representation of samples returned to the statistical analysis of the current study.

From the total of 182 patient samples (Supplementary Materials Table S2), 21 datasets had to be removed from the cohort, as their QC parameters were not within the test acceptance range (Figure A1), resulting in a total of 161 patient NMR data entries (Figure A1). A detailed description of the QC analysis is shown in Appendix A.

### 2.4. Univariate Statistics Identified High HDL-4 Parameters and Elevated LDL-2 Cholesterol in the AD Group Only

Univariate analysis of variance (ANOVA) identified four lipoprotein variables with notable changes with the acquired cohort lipoprotein dataset without a logarithmic normalization (*p* < 0.10, Supplementary Materials Figure S1). Herein, three variables belong to the HDL-4 subfraction, including Apo-A1 apolipoprotein, cholesterol, and free cholesterol. The fourth variable, LDL-2 subfraction cholesterol (L2CH), demonstrated a slight elevation in the AD group only. The observed changes suggest a transition to higher levels of H4A1, H4CH, and H4FC in the AD and MCI groups (Supplementary Materials Table S3).

The applied principal component analysis (PCA) scores plot demonstrates no group discrimination. The same was observed for the regression model analysis sparse Partial Least Squares Discriminant Analysis (sPLS-DA), where no separation of the 95% confidence intervals was found (Supplementary Materials Figure S1). Based on the loadings plot, however, three HDL-4 subfraction-based parameters were found (H4CH, H4FC, and H4A1) that were also notable by ANOVA (*p* < 0.10).

Finally, Partial Least Squares Discriminant Analysis (PLS-DA) identified further a contribution of several LDL subfractions and a total blood particle number (L1PN–L3PN, L6PN, and TBPN) in the regression model of three-group comparison of the lipoprotein dataset (Supplementary Materials Figure S2a). Herein, these described parameters together with total blood cholesterol (TPCH) and LDL fraction cholesterol (LDCH) has been found elevated in the AD group. ANOVA-highlighted lipoprotein entries of HDL-4 Apo-A1 apolipoprotein (H4A1) and LDL-2 cholesterol content (L2CH) have been listed. The extracted VIP score values were evaluated and shown in Supplementary Materials Table S3.

### 2.5. Intermediate-Density Lipoprotein (IDL) Triglycerides and Very Low-Density Lipoprotein VLDL Lipoprotein Parameters Are Higher in Dementia Groups via Volcano Plot Analysis That Includes Mini-Mental State Examination MMSE Scores and Age

**Figure 1:**
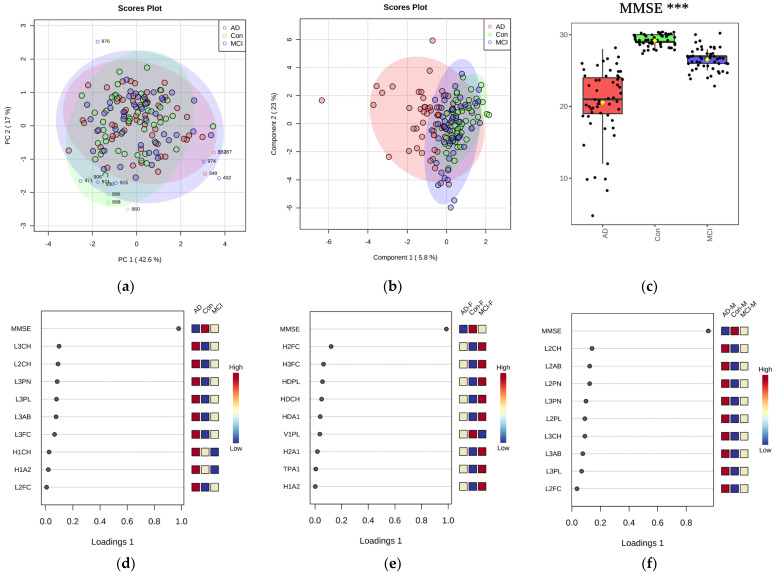
Multivariate analysis of combined NMR lipoprotein and clinical parameters plotted via PCA scores (Panel (**a**)), outliers shown), sPLS−DA regression model analysis scores plot (Panel (**b**)). The data displayed include the MMSE score and age (Panel (**c**)). Additionally, the significance of MMSE is further illustrated by the top 9 lipoprotein data variables ranked by component 1 significance of the sPLS−DA regression model analysis loadings plot (Panel (**d**)), which are L3CH, L2CH, L3PN, L3PL, L3AB, L3FC, H1CH, H1A2, and L2FC. sPLS−DA regression model analysis of three patient group comparisons separated by gender criterion is shown (Panel (**e**,**f**)). Significance: *** *p* < 0.001. F − female. M − male.

We were able to identify 10 lipoprotein variables with an ANOVA *p* < 0.10 (Supplementary Materials Table S4) namely LDL-3 subfraction (cholesterol, particle number, phospholipids, Apo-B100 apolipoprotein, free cholesterol), LDL-2 cholesterol, VLDL-2 free cholesterol, VLDL-1 triglycerides, IDL triglycerides, and HDL-4 subfraction Apo-A1 apolipoprotein. Next, we added the clinical MMSE score and age to the lipoprotein data and repeated the multivariate statistical analysis (Figure 1). We evaluated the direction of changes and the connection of lipoprotein parameters using a regression model with the introduction of the MMSE score to the statistical analysis. We found that HDL and LDL lipoprotein characteristics explained the difference between patient groups for this regression model analysis.

Disclosed in Appendix B are discrepancies in serum lipoprotein concentrations that were observed owing to a gender bias in the current study. The data analyzed were taken from the whole cohort dataset of lipoprotein values and after the QC assessment (Appendix A). The investigation showed that the female cohort contributed more HDL parameters, whereas the male patients and controls contributed more LDL parameters.

The prevalence of elevated HDL-4 subfraction levels was highest in female patients diagnosed with moderate cognitive impairment (MCI), followed by Alzheimer’s disease (AD). Male AD patients tended to have somewhat higher levels of LDL-2 cholesterol.

We were able to see a number of variables, previously highlighted from the ANOVA analysis, to be highly descriptive in a three-group regression model analysis via PLS-DA (Supplementary Materials Figure S2b). In that comparison, most contrasting changes have been shown by LDL-2 and -3 subfractions cholesterol levels, LDL-3 subfraction Apo-B100 apolipoproteins amount (L3AB) and statistically insignificant HDL-1 subfraction Apo-A2 apolipoprotein variables to be higher in AD. The parameter for VLDL-2 free cholesterol (V2FC) has been found to be lower in MCI.

In contrast to the PCA, the regression model sPLS-DA score plot demonstrates a partial cluster separation, which can be seen mainly as a result of including the MMSE parameter. For the current comparison, a logarithmic transformation was performed to minimize the magnitude effects of the different units. The box plot for MMSE shows a significance of *p* < 0.001.

Moreover, performing a PatternSearch analysis, several lipoprotein parameters correlated positively (IDL triglycerides, VLDL triglycerides including VLDL-1 and -2 subfractions, VLDL-1 and VDL-5 subfraction phospholipids, VLDL-1 subfraction cholesterol, VLDL apolipoproteins Apo-B100, VLDL particle number, HDL-3 and HDL-4 subfraction triglycerides) with the MMSE score and negatively with age (Supplementary Materials Figure S3). On the other hand, numerous parameters had inverse associations: multiple HDL-1 variables (H1CH, H1A1, H1PL, H1FC, and H1A2), HDL-2 subfraction cholesterol, and multiple HDL-2 variables (L2FC, L2CH, L2PL, L2AB, and L2PN).

### 2.6. Adding Cerebrospinal Fluid AD Biomarkers to the Lipoprotein Panels Identifies Increased Triglycerides and VLDL Fraction Variables in AD Samples

Using the established clinical CSF biomarkers Aβ and tau (Table 1), further insights into altered lipoprotein metabolism in single individuals could be obtained. Of note, such parameters were only available for the AD and MCI groups. Therefore, the control group was not used for that type of comparison.

**Figure 2:**
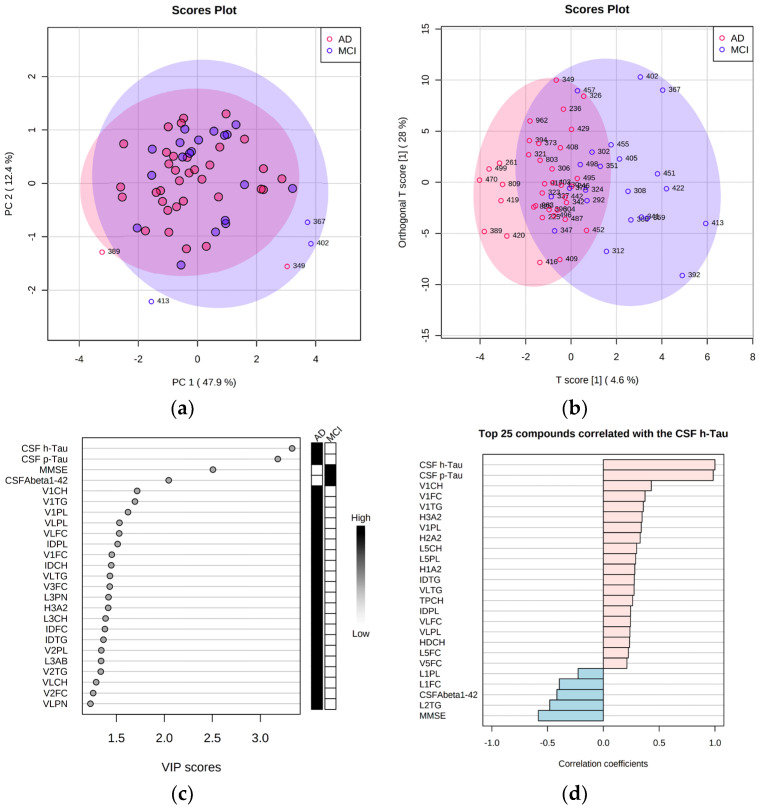
Multivariate analysis of 2−group lipoprotein and metadata comparison plotted via PCA scores plot (Panel (**a**), outliers shown), via the oPLS−DA regression model analysis scores plot (Panel (**b**)). Additionally, the top 25 data entries were plotted according to their variable importance (VIP) score based on the oPLS−DA regression model analysis ranked by T score (Panel (**c**)). Finally, a comparison of the top 25 Spearman correlations was performed in the context of CSF h−tau biomarker levels and visualized via a PatternSearch plot for a group of patients (n = 17) who showed the most apparent changes in the T score (Panel (**d**)).

Initial t-test-based volcano plot analysis (defined thresholds FC > 1.20, *p* < 0.10, VIP scores > 1.00, Supplementary Materials Table S5) showed that CSF biomarkers (Aβ, h-tau, p-tau) were in the highest significance range among all features. From the lipoprotein NMR parameters VLDL and IDL fraction variables showed significant elevations in the AD subselection (Figure 2). The regression model analysis (oPLS-DA) further indicated an AD-influenced imbalance of lipoprotein metabolism. The investigation (Supplementary Materials Figure S2c) showed that the lipoprotein parameters from VDLV fraction (VLDL-1 cholesterol, triglycerides, phospholipids, and free cholesterol; VLDL-2 and VLDL-3 free cholesterol) and IDL fraction phospholipids (IDPL) were elevated in the blood of AD patients compared against the MCI group.

Relying on T scores (Figure 2b), we identified a number of patients (n = 17, patients 261, 389, 419, 420, 470, 499, 809, 308, 341, 359, 367, 388, 392, 402, 413, 422, and 451) that were discriminated within the regression model analysis. Herein, LDL-2 triglycerides showed a significant Spearman correlation value of > 0.5 (Figure 2d).

### 2.7. Correlation of NMR Lipoprotein Data with Clinical Metadata Identifies Triglycerides as Key Variables for the Correlation with Aβ Levels and ApoE4 Status

For a comprehensive evaluation of the main NMR lipoprotein parameters with the clinical metadata (Supplementary Materials Table S6) we applied a correlation panel plot approach, as previously described in [24], and focused on the 31 main lipoprotein variables of the B.I. LISA^TM^ assay.

**Figure 3:**
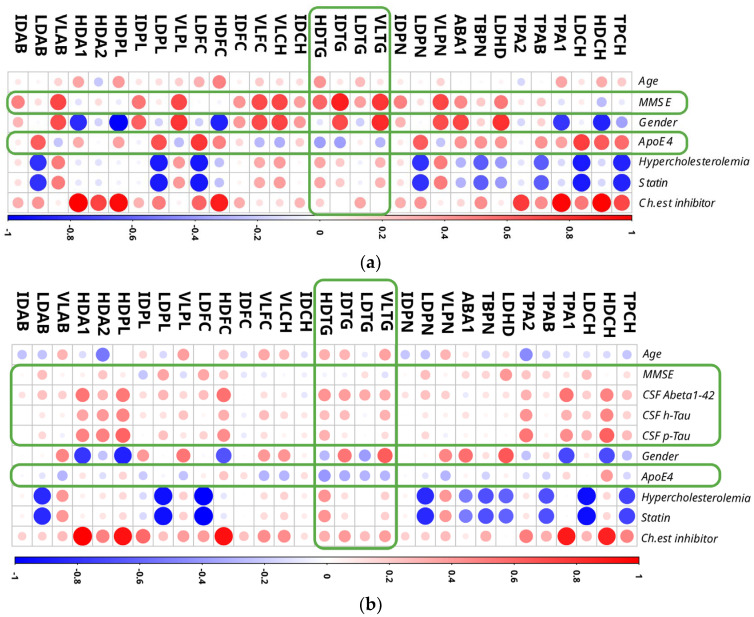
Correlation analysis of 31 lipoprotein variables (without subfractions) with clinical metadata. Three−group analysis (Panel (**a**)) and 2−group analysis (AD and MCI groups, Panel (**b**)) plots represent Spearman correlations that have been rescaled to fit the resulted plots. Shown correlations areas from −1 (blue) to 1 (red). In row ‘Gender’, red dots denote female patients, blue dots male patients. Frames in green represent the main finding of the analysis applied. Four triglyceride parameters among all lipoprotein fractions were strongly correlated with MMSE, CSF biomarkers, and ApoE4 status: HDTG, IDTG, LDTG and VLTG. Lipoprotein abbreviations are disclosed in Supplementary Materials Table S1. Ch.est inhibitor – cholinesterase inhibitor treatment status.

The Spearman correlations showed for ApoE4 a repeated pattern of the HDL, IDL, LDL, and VLDL triglycerides (HDTG, IDTG, LDTG and VLTG, Figure 3). In particular, these four triglyceride parameters correlated with the MMSE score (Figure 3a) and with Aβ and ApoE4 (Figure 3b). This is supported by Supplementary Materials Table S7 which also shows elevated VLDL and IDL triglyceride parameters when comparing MCI patients to controls. Therein, the regression model analysis also provided high discriminative values to HDL-4 lipoprotein variables (H4A1, H4PL, H4FC, H4CH) that were higher in the MCI group (Supplementary Materials Figure S2d).

The Spearman correlations for statin therapy for patients with high blood cholesterol levels (hypercholesterolemia) and cholinesterase inhibitor treatment (“Ch. est. inhibitor”) to improve AD-dementia health conditions revealed intriguing connections. Positive associations were observed for VLDL particle number and apolipoprotein Apo-B100 variables in the case of hypercholesterolemia. Total blood cholesterol (including LDL), LDL-free cholesterol, total blood apolipoprotein Apo-B100 (including LDL), Apo-B100/Apo-A1 apolipoprotein ratio, LDL/HDL lipoprotein ratio, total blood particle number (including LDL), and LDL phospholipids, on the other hand, were found to be negatively associated with the given metadata. Interestingly, all of the currently mentioned lipoprotein data variables had the indicated relationships with statin and hypercholesterolemia data in both cohorts evaluated (Figure 3). Finally, for cholinesterase inhibitor medication therapy, we found positive relationships with total blood, HDL and LDL cholesterol levels, total blood Apo-A1 and Apo-B100 apolipoprotein levels, HDL and LDL free cholesterol levels, HDL phospholipids and apolipoproteins (ApoA1 and ApoA2). The matching components between the two comparisons (Figure 3) were HDL cholesterol and free cholesterol, total blood and HDL apolipoprotein Apo-A1, and HDL phospholipids.

From the correlations presented in Figure 3, we could observe several considerable connections between the gender (red dots = correlation with female, blue dots = correlation with male) of a patient and lipoprotein parameters like LDL/HDL lipoprotein, Apo-B100/Apo-A1 ratio, VLDL and IDL triglycerides, VLDL cholesterol and free cholesterol, VLDL Apo-B100 apolipoprotein and VLDL phospholipids. For the male patients of the study, we identified full cohort correlations that indicated higher levels of HDL-free cholesterol, phospholipids and Apo-A1 apolipoprotein content.

Interestingly, a different situation is shown at the AD-MCI comparison (Figure 3). Therein, the higher levels of HDL cholesterol, total blood Apo-A1 apolipoprotein amount, HDL-free cholesterol, HDL phospholipids and HDL Apo-A1 apolipoproteins were found for the male population. From the both correlational plots it could be concluded that, to some extent, the metadata factors–gender and choline esterase inhibitor treatment were found symmetrically correlating with the analyzed NMR lipoprotein dataset values.

### 2.8. Gender-Specific Alterations of HDL and VLDL Lipoprotein Parameters Are Mostly Characteristic for AD Patients and Only to a Little Extend for MCI Subjects

Appendix B summarizes which lipoprotein parameters were changed within a patient group under of consideration of gender. Hereby, lipoprotein features were more significant in the AD comparison than MCI, with basically no gender-specific alterations in the Control group (Table A1, Con).

**Figure A2:**
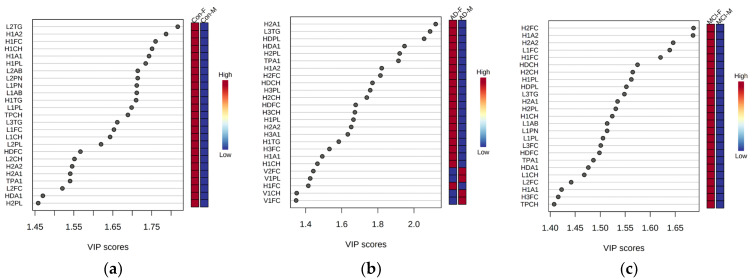
Multivariate analysis of the three male-female group comparisons based on oPLS-DA ((Panel (**a**)), in the cognitively control group; (Panel (**b**)), AD patients; (Panel (**c**)), MCI patients). The score plots include the top-25 lipoprotein variables ranked by the highest importance score. F–female. M–male.

The MCI groups of male and female patients were mostly discriminated by the following HDL and LDL features which all showed low FDR-adjusted *p*-values and high regression VIP score values (Table A1, MCI, and Figure A2): HDL-1 subfraction Apo-A2 and Apo-A1 apolipoproteins, phospholipids, cholesterol, and free cholesterol; HDL-2 subfraction free cholesterol, total HDL cholesterol, and phospholipids; and LDL-1 and LDL-2 free cholesterol.

**Figure A3:**
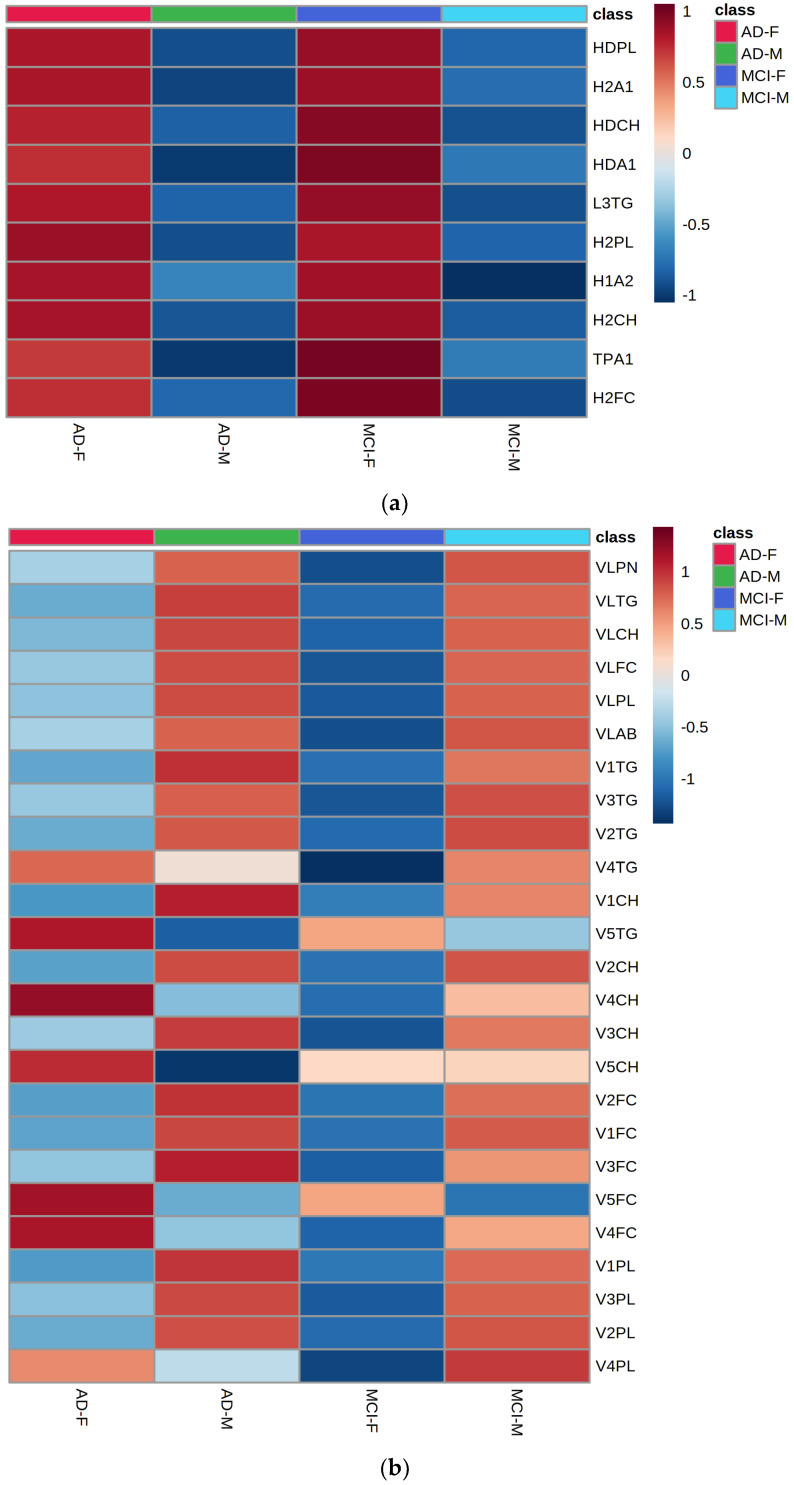
Heatmap plot analysis based on male−female comparisons within patient groups (AD, Con, and MCI) for the dataset from the whole cohort lipoprotein data and a selection of the top−10 features for females (Panel (**a**)) alongside illustration of the VLDL parameters that were high in male groups (Panel (**b**)).

Considerably more significant differences were seen when comparing females against males in AD subjects only (Table A1 and Table A2, AD, and Figure A2 and Figure A3). Those were for females’ alterations in the HDL-2 subfraction, Apo-A1 apolipoproteins, phospholipids, and cholesterol, LDL-3 triglycerides with HDL phospholipids changes, HDL Apo-A1 and HDL cholesterol. In addition, for males, some VLDL characteristics were close to the FDR < 0.05 significance threshold, namely VLDL-1 subfraction cholesterol, phospholipids, and triglycerides, as well as VLDL-2 free cholesterol and VLDL-5 triglycerides.

From a statistical perspective, the *p*-values provided above were the most significant determined within this study. However, we want to emphasize that these findings are not necessarily attributed to dementia but can also be a result of confounding factors such as comorbidities or disease-associated change of lifestyle and diet.

## 3. Discussion

Using a commercially available quantitative lipoprotein assay based on 600 MHz IVDr NMR spectroscopy, we were able to correlate a set of 112 lipoprotein variables with clinical metadata and established AD biomarkers in CSF to obtain deeper insights into the pathophysiology of dementia and elucidate novel potential diagnostic AD serum markers.

The most prominent findings of our present study were newly found increases in HDL-4 variables (ApoA1 apolipoprotein, free cholesterol, and cholesterol) in both dementia groups and changes in triglycerides (VLDL, including VLDL-1 and VLDL-2, and IDL) that were higher in AD specimens. Furthermore, we discovered VLDL-1 and LDL-2 cholesterol content alterations that were particularly high in the blood of elderly with AD.

These observations allowed us to construct a refined picture of serum lipid metabolism in the studied cohorts. It is well known that lipoprotein parameters, such as LDL cholesterol, are associated with dementia and cardiovascular disease [6,19]. For instance, LDL cholesterol is heavily influenced by dietary routines and body weight in the elderly [25,26] and therefore offers an easy target for therapeutic intervention, e.g., by adapted nutrition.

However, using only a limited set of lipoprotein parameters alongside a large variety of metadata confounders (age, gender, physical activity, etc.) limits the use of such parameters for diagnosis and therapy monitoring. In contrast, using an extended lipoprotein panel of hundreds of subclass parameters is much better suited for personalized precision medicine and patient stratification.

Our findings demonstrate that NMR-based lipoprotein variables—when absolutely quantified—can provide such knowledge and show high dispersion rates within AD, MCI, and controls, as supported by the distinguished MMSE scores in the patient groups.

**Figure 4:**
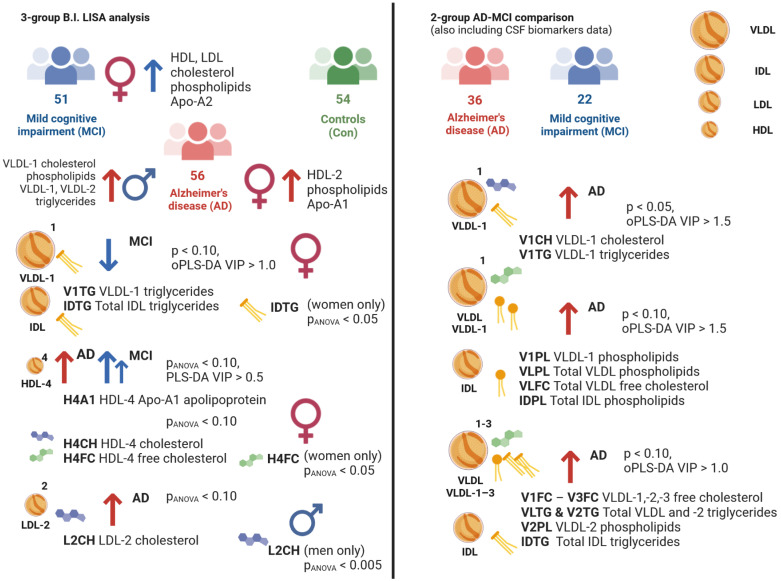
Graphical summary of the results presented in this study.

Within the full cohort comparison, the NMR lipoprotein data showed several notable changes (Figure 4). Starting from the two-group comparison MCI-Con, we observed lower levels of VLDL-1 and IDL triglycerides in the serum of mild cognitive impairment patients. This is a particularly interesting finding, as multiple reports suggest lower levels of total blood triglycerides in this patient group [4,5,6], and we were able to identify similar observations as reported previously [25]. Moreover, we were able to identify a considerable correlation between VLDL triglycerides and MMSE scores alongside CSF Aβ values.

Our investigation further identified a combination of HDL-4 subclass parameters, which was significantly elevated in both AD and MCI. Among them, HDL apolipoprotein Apo-A1 indicates a vital link between HDL synthesis and cholesterol delivery from the liver [26]. This is very important, as Apo-A1 levels have recently been discovered to be significantly higher in the PD-dementia group of MCI patients [27]. We suggest the following lipid homeostasis dysregulations based on the current findings: changes in HDL lipid traveling through the blood–brain barrier [28,29], its integrity [30], and the amyloid plaque formation process [31].

Interestingly, we found group-specific changes in our study that affected only the HDL-4 subfraction (H4A1, H4CH, H4FC) and, therefore, indicated a specific stage in ongoing cognitive decline. These three parameters suggest an increased accumulation in the serum of the MCI group and only a slight elevation in the AD group. Similar to a previously reported study [6] on HDL cholesterol and dementia, our findings on H4A1, H4CH, and H4FC remain the key findings of this study. HDL cholesterol and free cholesterol are potential indicators of cholesterol excess in the AD brain due to their leakage through the blood–brain barrier [32].

As mentioned above, we identified a widely known AD-descriptive parameter, LDL cholesterol, to be significantly changed in the AD group [33]. In the current study, we were able to identify the subfraction LDL-2, for which cholesterol levels showed an elevation, as a specific parameter among the LDL cholesterol variables. It has been reported that the LDL-2 cholesterol parameter is closely correlated with VLDL cholesterol, IDL cholesterol, and LDL-1 cholesterol during statin treatment [34]. Another study revealed a slight tendency of lowered LDL cholesterol in subjects with MCI compared to AD [19].

When a subcohort of AD and MCI patients was compared, VLDL-1 parameters and, importantly, a statistically significant increase in VLDL-1 triglycerides in the AD group may be correlated with a prediabetic condition. Individuals with insulin resistance have a higher production of VLDL particles, which are responsible for triglyceride transport [35]. Of note, the VLDL-1 subfraction is the most numerous and carries the majority of serum triglycerides.

In the context of AD and the close interconnection of blood lipoprotein biomarkers of type 2 diabetes and AD, with some groups referring to AD as “diabetes type 3” [36], we could conclude that the increased VLDL-1 triglycerides provides a high degree of importance within the current investigation. With the given evidence that the VLDL-1 cholesterol (V1CH) parameter is generally elevated in the serum of subjects with ApoE4 positive status [37], we were able to detect V1CH in significantly higher amounts in the AD group.

We also discovered numerous VLDL entities that were higher on average in AD patients. The link between a high cholesterol dietary intake and Aβ aggregation has been known for some time [38]. One of the factors with a major influence on Aβ levels is the apolipoprotein E type present in a system. When a patient shows altered ApoE4 function, AD-related risks increase multiple-fold [39]. In general, ApoE affects the balance of VLDL lipoproteins in blood [40]. The liver-generated very-low-density lipoproteins operate in a preferential binding of ApoE type 4, caused by domain interactions [41]. ApoE4 levels and hence cholesterol transport levels have an impact on cholesterol homeostasis and neural plasticity. Based on the findings of the current study and a literature search, we think that there is a link between total cholesterol, ApoE4, several VLDL parameters, and amyloid plaque formation. From this perspective, the total cholesterol elevation in patients’ blood is one of key factors indicating AD-driven dementia risk [42].

Interestingly, AD-induced elevation of VLDL free cholesterol was reported previously [43]. Herein, the authors reported that the IDL fraction of phospholipids was significantly increased in AD patients, which was reproduced in our investigation results [43]. This variation warrants future investigations, as IDL phospholipids were also found to be elevated in a type 2 diabetes drug treatment study, suggesting a restored lipid metabolism in diabetes patients [44]. The observed cholesterol alterations between AD and MCI based on free and esterified forms of cholesterol, as hypothesized in [45], are believed to be one of the factors defying lipid dysregulation within our studied cohort.

Overall, the observations made in the subcohorts of AD and MCI indicate changes closely related to predisposing factors of amyloid aggregation. The VLDL lipoprotein imbalance, therefore, presents evidence for lipid metabolism alterations in the serum of dementia patients.

We discovered a tendency toward a positive connection between serum triglycerides and CSF Aβ levels. Among the lipoprotein class data, it has been previously known that AD-driven dementia risk is linked to blood triglyceride levels [32]. We suggest that the observed results provide a chance for further evaluation of cognition status alongside serum triglycerides in future investigations.

The triglyceride imbalance in the serum of AD patients may be indicative of the existing amyloid pathology in the brain. Furthermore, triglycerides seem to be closely associated with ApoE4 status with similar intensity to that described for CSF Aβ levels in AD [46].

The examination of the resulting impact of statin therapy for hypercholesterolemia revealed that cholesterol levels were actually reduced, particularly in the LDL fraction, which is an aftereffect of known processes involving ApoE apolipoproteins that transport cholesterol molecules [47]. In [48], researchers discovered a link between LDL and HDL lipoprotein fraction levels, which can be compared as an LDL/HDL ratio, and higher blood cholesterol levels and poor attention performance test results in patients with type 2 diabetes. We discovered a relationship between the LDL/HDL ratio and cholesterol levels, and they all exhibited a negative correlation with statin medication status. Another AD indicator is the apolipoprotein Apo-B100, which has been demonstrated in our study to be reduced throughout statin therapy. This shift was also observed in a study of Alzheimer’s and MCI patients [49]. Because Apo-B100 is predominantly found in LDL and VLDL, it was quite noteworthy to observe that the two metrics VLAB and LDAB were inversely related to statin therapy status. Meanwhile, one study showed that concentrations of Apo-B and p-Tau in the presymptomatic phase of AD patients with a family history of Alzheimer’s disease are likely to correlate with a risk of vascular or mixed dementia [50].

From the standpoint of cholinesterase inhibition therapy, we could only see an increase in HDL cholesterol (HDCH, HDFC), HDL phospholipids, and Apo-A1 apolipoproteins alongside the therapy status. In this case, the HDL-cholesterol transporter Apo-A1 is restored in blood after therapy, as evidenced by our data. Further on from the correlational analysis, we were able to detect a close interaction between the fact of the therapy and a gender of the patient. Previously, it had been already brought to a discussion whether cholinesterase inhibition therapy alters brain functionality in different ways for dementia patients based on their sex [51,52]. Thus, our findings represent a novel piece of evidence in that context. Future research should investigate in-depth how cholinesterase inhibition affects therapy changes amongst dementia-suffering subjects and how NMR-based lipoprotein profiling potentially could be used hereby as monitoring tool.

We found significant alterations of NMR parameters related to AD, such as HDL fraction lipoproteins to be higher in MCI female patients, while LDL fraction was higher in the group of AD male subjects. The HDL-4 subfraction was the only one in females that showed to be a valid dementia discriminatory factor, while LDL-2 cholesterol was the key factor for the male sub-cohort.

In addition, we identified gender-specific differences mostly in AD-dementia subjects, even though mild variations of the same parameters still were seen in the MCI comparison. By contrast, the cognitively healthy controls displayed no statistically significant variations in lipoproteins when comparing females and males.

Intriguingly, one research study discovered that female and male variations in HDL2-, HDL-3, HDL cholesterol, and Apo-A1 apolipoprotein characteristics are relatively comparable [53]. The same study reports that HDL Apo-A1 parameters were higher in AD-female counterparts, while MCI-type dementia in elderly women was characterized by raised HDL and LDL cholesterol. In our study, we found higher LDL-3 triglycerides in female AD patients, which has to be taken within the context that triglycerides in blood are susceptible to change in relation to gender and AD-onset [54]. For our cohort, apolipoprotein Apo-A2 showed a significant elevation in MCI women. In addition to that, decreased Apo-A2 levels in the MCI male group might also be attributed to the males’ lipidomic response towards dementia, as previously discovered [6]. Other novel findings were elevated HDL-2 phospholipids in AD females and LDL-3 triglycerides in MCI females. Finally, discovered yet significant by multiple hypothesis testing results of multiple VLDL lipoprotein fraction parameters that have been significantly higher in AD men may be correlated to the covariates, e.g., body weight [55]. Therefore, the significance of VLDL in the gender comparison was lower in contrast to parameters described earlier. Overall, the obtained gender-based findings suggest even deeper investigation into the influence of sex and comorbidities when investigating dementia and other neurodegenerative diseases [56].

We would like to stress that even though many of the solely gender-based comparisons showed strong FDR values, these findings are prone to multiple confounding factors, such as smoking [57]. Other comorbidities, such as diabetes type 2, cardiovascular disease, imbalances in dietary supplementation, obesity, and various other health conditions, including the emerging coronavirus pandemic, can further alternate the lipoprotein profile on a long-term basis [58,59,60]. Therefore, we may once more address the rather insignificant main findings of the dementia-driven changes where we estimate that larger cohorts with much higher n numbers and consideration of clinical metadata that were not available within this study would have resulted in stronger statistical findings.

In conclusion, triglycerides and other lipoprotein parameters, mainly within the VLDL and IDL lipoprotein fractions, seem to be potentially useful discriminators for the stratification of AD patients. These features could be successfully identified using a quantitative NMR-based approach which is a proof of concept that the applied assay can become an important add-on to the AD diagnostic toolbox in the near future. However, the use of a larger n number of patient specimen deriving from bigger cohorts or by adding existing data, alongside the full consideration of clinical metadata, is necessary, before the identified features within this study can be fully evolved and validated as novel AD and MCI-like biomarkers and qualify their use for a refined stratification of dementia patients.

## 4. Materials and Methods

### 4.1. Study Design

The participants were selected from the biofluid biobank at the Hertie Institute for Clinical Brain Research, the Center of Neurology, University of Tübingen, Tübingen, Germany. The participants were divided into different phenotypes as illustrated in Table 1 according to their clinical diagnosis and their CSF biomarkers for AD: Aβ1-42 (> 800 pg/mL threshold), h-Tau (< 300 pg/mL threshold), and p-Tau (< 60 pg/mL threshold). CSF samples were available for 43.1% of the MCI individuals and 64.3% of the AD participants.

Additional metadata parameters were received and considered for analysis: age, gender status (0–male, 1–female, for the purpose of categorization within the statistical software), mini-mental state examination (MMSE) score, cholinesterase inhibitor drug treatment positivity status (chol-est inh), number of ApoE4 alleles, hypercholesterolemia and statin drug treatment status.

The experimental protocols described in the present study have been approved by the ethic committee of the medical faculty of Eberhard Karls University of Tübingen (Tübingen, Germany) and University Hospital Tübingen, Germany (protocol code 721/2015BO2, 07.08.2019, SOP-protocol Biobank HIH-Biobank version 1.2.5, February 2020). All methods were performed in accordance with the relevant guidelines and regulations. All participants provided written, informed consent.

### 4.2. Sample Collection

A total of 182 blood samples were collected into standard serum container tubes. Whole blood was taken for serum preparation in tubes containing a clot activator and allowed to coagulate for 30 min at room temperature. The serum was then removed from the clot using conventional procedures. All serum aliquots were frozen and stored at −80 °C until analysis.

### 4.3. NMR Sample Preparation and Experiments

Serum aliquots were stored at −80 °C and transported on dry ice until preparation for NMR analysis. All samples were prepared according to a commercial in vitro diagnostics research (IVDr) NMR SOP for blood serum (AVANCE IVDr Methods Version 003, Bruker BioSpin GmbH, Ettlingen, Germany). On the day of preparation, the samples were thawed at room temperature and then directly prepared. Serum samples (350 μL) were mixed 1:1 with a pH neutral (pH = 7.40) plasma/serum preparation buffer (350 μL, order number AH0622-10, provided by Bruker BioSpin GmbH, Ettlingen, Germany) containing 20% deuterium oxide, 0.075M sodium monophosphate, 0.08% sodium 3-(trimethylsilyl)-2,2,3,3-tetradeuteropropionate (TSP) and 4% bacteriostatic sodium azide, as published previously [61].

After thoroughly mixing without vortexing, an aliquot of 600 µL from the resulting mixture of serum and serum buffer was transferred into a 4” 5 mm NMR glass tube (order number Z168405) and then placed into an autosampler (Bruker SampleJet™, Bruker BioSpin GmbH, Ettlingen, Germany). Samples were stored at 6 °C in the autosampler prior to analysis and kept for 5 min inside the NMR probe head to reach temperature stability.

NMR experiments were accomplished on a Bruker Avance III HD 600 MHz NMR spectrometer (Bruker BioSpin, Fällanden, Switzerland). Samples were measured with a 5 mm TXI probe using Bruker TopSpin version 3.6.1, including additional required IVDr experiments and software plug-ins as provided by Bruker BioSpin GmbH (Bruker BioSpin, Ettlingen, Germany). Quality control (QC) was performed regularly and was accomplished within the analysis package Bruker IVDr BioBank QC B.I.BioBankQC™ (Bruker BioSpin, Ettlingen, Germany).

Serum spectra were recorded using ^1^H NOESY (nuclear Overhauser effect spectroscopy) experiments at 310.0 K. ^1^H NOESY spectra were utilized within the provided Bruker IVDr Lipoprotein Subclass Analysis B.I. LISA™ to generate a prepared dataset for automated metabolite annotation and quantification (provided by Bruker BioSpin GmbH, Ettlingen, Germany). The parameters are listed in Supplementary Materials Table S1. Lipoprotein subfractions are numbered in increasing density, such as VLDL-1 to VLDL-5, as previously documented in [62,63,64,65].

### 4.4. Statistical Analysis

Statistical analysis was performed with the MetaboAnalyst 5.0 package (Xia Research Group, McGill University, Montreal, Canada) [66]. As different concentration units for combined analysis were used, the dataset was additionally logarithmically transformed when adding information on MMSE, CSF biomarkers, and clinical metadata. Observed 85 value cells from the lipoprotein dataset that had a zero value were replaced with an empty cell and then underwent the KNN missing value estimation within the MetaboAnalyst. Data from the CSF biomarkers and other metadata were normalized identically to the lipoprotein data. For each comparison, the following set of parameters was determined: *p* values (Student’s t-test and one-way ANOVA, analysis of variance), Spearman correlation coefficients (used in PatternSearch plots), PCA (principal component analysis) loadings, oPLS-DA (orthogonal partial least square discrimination analysis), sPLS-DA (sparse partial least square discrimination analysis), and VIP (variable importance in projection) scores of the metabolites. To produce consistent statistical tests, we applied the following set of thresholds: threshold *p* < 0.10, VIP scores threshold for robust group discriminator findings VIP > 1.00. Additionally, to address multiple hypothesis testing, we calculated the false discovery rate (FDR) of each variable that showed a *p*-value < 0.1. One-way ANOVA and post-hoc tests were used (e.g., AD-MCI, Con-MCI, etc.) with the Fisher’s method used for FDR calculation. When FDR values do not pass the < 0.1 threshold, high VIP scores can be used as indicator that the corresponding variable still is a promising feature to distinguish between distinct groups. Accordingly, we listed in all tables raw *p*-values, FDR *p*-values and respective VIP scores. Correlational analysis plots were produced with the use of the free software environment R (R version 3.6.2, R Core Team) package “corrplot”. Finally, a graphical representation of the results in the figures was created with the BioRender.com online-based service with the use of relative scaling of lipoproteins, as shown in [67].

## 5. Conclusions

Using an average-sized clinical patient cohort, we discovered a relationship between the cognitive phenotype of AD, AD biomarkers in CSF and serum lipoproteins as measured by quantitative IVDr NMR spectroscopy. VLDL parameters, including triglycerides and IDL triglycerides, were increased in AD. LDL-2 subfraction cholesterol showed a modest increase in AD and a higher HDL-4 subfraction in both AD and MCI.

Three variables (HDL-4 Apo-A1 apolipoproteins, cholesterol, and free cholesterol) showed alterations that indicated a shift to higher levels in MCI before AD. This suggests that there is a critical relationship between HDL production and cholesterol transport from the liver, which is this study’s principal result. From the conducted gender-based comparisons, we identified that HDL-4 parameters were only altered in the serum of female subjects. At the same time, LDL-2 cholesterol was higher in male patients suffering from AD. Moreover, we identified several correlations between the patients’ gender and status of the cholinesterase inhibition therapy via the analysis of the presented lipoprotein data. Investigating gender-based differences between the AD and MCI patients solely (not compared to controls), we found in female patients FDR-significant elevations of HDL and LDL cholesterol, HDL apolipoprotein Apo-A1, HDL phospholipids, and LDL triglycerides. Fairly smaller by significance, various VLDL parameters were found elevated among AD male subjects.

Further on, VLDL lipoprotein and several triglyceride values were on average higher in AD patients. Correlations between VLDL, triglycerides and CSF Aβ biomarker levels suggest an AD-influenced imbalance of lipoprotein metabolism.

The presence of high HDL-4 (especially in females) and triglyceride levels in the elderly is a proof of concept that this NMR-based lipoprotein assay is able to potentially differentiate AD from other diseases monitored, especially when cohort sizes are large. The quantification and standardized nature of the analytical approach also promote using the published data to increase n numbers of smaller cohorts to form super cohorts for in-depth investigations of altered lipid metabolism in AD and further neurological diseases.

## Figures and Tables

**Table 1:** Clinical data of the study cohort including readings of CSF biomarkers (Aβ1-42, h-tau, p-tau) for the three studied groups–Alzheimer’s disease (AD) and mild cognitive impairment (MCI) diagnosed patients, and control samples (Con).

	Total	Controls (Con)	Mild Cognitive Impairment (MCI)	Alzheimer’s Disease (AD)
patients	161	54	51	56
female	71	29	17	25
male	90	25	34	31
age (mean ± SD)	70.2 ± 7.4	70.4 ± 4.5	69.8 ± 8.6	70.5 ± 8.5
MMSE (mean ± SD)	25.3 ± 4.8	29.2 ± 0.8	26.5 ± 1.5 ***	20.5 ± 5.0 ***
CSF biomarkers J123Marija (Aβ, h-tau, p-tau) measured				
patients	58		22	36
female	24		7	17
male	34		15	19
age (mean ± SD)	68.7 ± 8.5		68.2 ± 9.3	69.0 ± 8.0
MMSE (mean ± SD)	23.4 ± 4.3		26.5 ± 1.8	21.5 ± 4.3 ***
CSF biomarker Aβ (pg/mL, mean ± SD)	619 ± 287		751 ± 378	538 ± 177 **
CSF biomarker h-tau (pg/mL, mean ± SD)	681 ± 356		503 ± 260	790 ± 366 ***
CSF biomarker p-tau (pg/mL, mean ± SD)	86 ± 33		69 ± 30	96 ± 31 ***
ApoE4 alleles				
measured (mean ± SD)	0.49 ± 0.62	0.40 ± 0.57	0.39 ± 0.57	0.70 ± 0.68 **
patients	152	53	49	50
female	68	29	16	23
male	84	24	33	27
age (mean ± SD)	70.2 ± 7.3	70.3 ± 4.5	69.9 ± 8.7	70.5 ± 8.3
MMSE (mean ± SD)	25.6 ± 4.4	29.2 ± 0.8	26.6 ± 1.5 ***	21.0 ± 4.4 ***

Statistical significance: ** *p* < 0.01, *** *p* < 0.001. SD–standard deviation. MMSE–mini-mental state examination. CSF–cerebrospinal fluid.

## Data Availability

Data is contained within the article or Supplementary Material. Fully anonymized individual quantitative NMR lipoprotein and QC reports are available upon request.

## Supplementary Materials

The following supporting information can be downloaded at: https://www.mdpi.com/article/10.3390/ijms232012472/s1.

## Appendix A

To evaluate the integrity of acquired NMR spectral data a built-in toolset Bruker IVDr BioBank QC B.I. BioBankQC™ was used. The quality control parameters cover related tests that must be completed in order to demonstrate that the NMR parameters applied to the sample are compatible with the parameters specified in the B.I. Methods.

From originally received 182 serum aliquots (Figure A1), the QC analysis was able to filter out 132 patient samples that had not reached critical check levels that endanger the sample’s stability, data readability, and analytical reproducibility. However, we later focused on reevaluating the remaining samples that had one considerable QC test that had not passed over the threshold levels of the tested variables.

Starting from the LineWidth (LW) test that describes overall NMR spectral quality, based on the given range of 1.5–1.7 Hz 8 samples were returned to the statistical analysis. The addition of increased LW parameter samples was performed on recognized influencing variables such as sample condition, patient fasting, and diet routine at the time of sample collection [68]. Therefore, the increase in the applied LW threshold was applied.

The second reevaluation resulted in one sample. It showed an elevated acetic acid concentration. We believe that acetic acid and several other metabolic variables (e.g., formic acid and lactic acid) that describe the sample’s microbiological activity must indicate the mentioned effect on several parameters at the same time. That led to samples returning to the analysis based on a possible individual increased acetic acid level in the patient’s serum. Finally, several matrix contamination test miss-check was a technical limitation of the QC. Finally, several matrix contamination test miss-check was a technical limitation of the QC. The limit of quantification (LOQ, 0.015 mmol/L) was not an optimal threshold for the quality performance test verdict. We, therefore, raised the threshold to 0.020 mmol/L. It could now correctly address the truly abnormal accumulation of the mentioned parameters.

## Appendix B

We also looked at the serum lipid profile considering only gender comparisons and not disease status and therefore checked by regression analysis the VIP scores for Controls, AD and MCI only (Figure A2).

We are able to observe major drifts of lipoprotein parameters with regard to the patient’s gender. Some changes had been seen as common between the three groups of the studied cohort. Notably, from the VIP scores plot ranked variables of HDL Apo-A1 apolipoprotein (H1A1, H2A1) was on average higher concentrated in the blood serum of female individuals. Such a finding has been already reported and referred to the taken diet [69].

In the current study, we could highlight the following HDL parameters: HDL-1 phospholipids (H1PL) and cholesterol (H1CH), also including LDL-2 triglycerides. AD-related abnormalities in male and female patients were studied, additionally it could be noted that in the MCI patient group there were elevated HDL cholesterol lipoprotein levels [70]. Finally, the similar variables (HDL-1 cholesterol) were observed in our cohort to be higher in the blood of cognitive control women.

From the statistical overview of the current findings (Table A1), we were able to further identify a significant elevation of HDL phospholipids in AD female subjects. This effect could be interpreted as HDL-driven AD progress even though such an alteration has been also reported for diabetic conditions [71,72].

Within a four-group analysis of female/male AD/MCI, the top 10 parameters were all evaluated in female patients (Figure A3a) while male patients generally showed higher VDL feature (Figure A3b).

**Table A1:** Volcano plot (FC > 1.20, *p* < 0.1) variables including false discovery rates (FDR) adjusted *p*-values and regression analysis VIP scores based on the male-female comparison of only AD patients (A), only Controls (B) and only MCI (C) patients.

A Variable, AD	*p* Value, AD	*p* (FDR Adjusted), AD	VIP (oPLS-DA), AD
H2A1	5.87 × 10^−6^	4.88·× 10^−4^	2.114
HDPL	8.80·× 10^−6^	4.88·× 10^−4^	2.047
L3TG	1.44·× 10^−5^	4.88·× 10^−4^	2.062
H2PL	1.76·× 10^−5^	4.88·× 10^−4^	1.913
HDA1	2.31·× 10^−5^	5.13·× 10^−4^	1.935
H2CH	6.55·× 10^−5^	0.0010	1.724
HDCH	1.01·× 10^−4^	0.0014	1.750
HDFC	1.80·× 10^−4^	0.0021	1.666
H3CH	1.97·× 10^−4^	0.0021	1.524
H3PL	2.05·× 10^−4^	0.0021	1.754
H1A2	2.67·× 10^−4^	0.0025	1.823
H1PL	2.89·× 10^−4^	0.0025	1.662
H3A1	3.83·× 10^−4^	0.0030	1.629
H2FC	5.14·× 10^−4^	0.0038	1.808
H1A1	8.89·× 10^−4^	0.0062	1.490
H1CH	0.0010	0.0067	1.458
H1TG	0.0014	0.0084	1.578
H2A2	0.0016	0.0091	1.662
H1FC	0.0025	0.0140	1.414
H3FC	0.0037	0.0196	1.524
V1CH	0.0047	0.0239	1.327
V1PL	0.0054	0.0263	1.411
V2FC	0.0068	0.0316	1.428
V5TG	0.0116	0.0495	1.206
V1TG	0.0116	0.0495	1.323
LDHD	0.0127	0.0523	1.157
V1FC	0.0156	0.0617	1.338
V5CH	0.0255	0.0976	1.193
VLTG	0.0264	0.0978	1.214
V3FC	0.0306	0.1096	1.066
V2PL	0.0341	0.1160	1.263
H2TG	0.0466	0.1477	1.040
V2TG	0.0517	0.1571	1.218
V3PL	0.0524	0.1571	1.042
VLPL	0.0655	0.1912	1.020
V2CH	0.0713	0.2030	1.021
IDTG	0.0755	0.2079	0.973
HDTG	0.0769	0.2079	1.003
V3CH	0.0787	0.2079	0.827
VLCH	0.0953	0.2301	0.831
**B Variable, Con**	***p* Value, Con**	***p* (FDR Adjusted), Con**	**VIP (oPLS-DA), Con**
H1CH	0.0181	0.3750	1.755
L2PL	0.0241	0.3750	1.601
L2AB	0.0278	0.3750	1.682
L2PN	0.0279	0.3750	1.682
H2CH	0.0306	0.3750	1.465
L2CH	0.0312	0.3750	1.530
H1PL	0.0317	0.3750	1.733
L2FC	0.0377	0.3750	1.532
H1A1	0.0483	0.3750	1.710
H1FC	0.0508	0.3750	1.766
H1A2	0.0636	0.3750	1.772
**C Variable, MCI**	***p* Value, MCI**	***p* (FDR Adjusted), MCI**	**VIP (oPLS-DA), MCI**
H1A2	9.37·× 10^−5^	0.0073	1.671
H1PL	1.85·× 10^−4^	0.0073	1.548
H1CH	2.44·× 10^−4^	0.0073	1.509
H1FC	3.50·× 10^−4^	0.0073	1.607
H2FC	3.82·× 10^−4^	0.0073	1.675
HDCH	3.96·× 10^−4^	0.0073	1.563
L1FC	5.99·× 10^−4^	0.0086	1.627
H1A1	6.19·× 10^−4^	0.0086	1.408
HDPL	8.08·× 10^−4^	0.0098	1.541
L2FC	0.0010	0.0098	1.430
H2A2	0.0011	0.0098	1.632
H2CH	0.0012	0.0098	1.551
L2PL	0.0012	0.0098	1.393
H2A1	0.0014	0.0098	1.524
L1AB	0.0014	0.0098	1.505
L1PN	0.0014	0.0098	1.504
H2PL	0.0016	0.0098	1.518
L3TG	0.0016	0.0098	1.538
HDFC	0.0021	0.0111	1.486
L1PL	0.0021	0.0111	1.496
L2AB	0.0025	0.0116	1.348
L2PN	0.0025	0.0116	1.347
L1CH	0.0027	0.0120	1.460
L2CH	0.0031	0.0132	1.270
L3FC	0.0041	0.0167	1.490
H3FC	0.0061	0.0235	1.392
LDFC	0.0088	0.0326	1.380
L3PL	0.0091	0.0326	1.283
L3PN	0.0109	0.0358	1.224
L3AB	0.0110	0.0358	1.224
V3PL	0.0123	0.0389	0.967
L2TG	0.0128	0.0394	1.378
H4TG	0.0135	0.0405	0.906
V2FC	0.0139	0.0405	1.069
V3CH	0.0158	0.0437	0.815
V3FC	0.0161	0.0437	0.906
L3CH	0.0178	0.0463	1.137
V2PL	0.0179	0.0463	1.028
V1PL	0.0210	0.0518	1.099
H1TG	0.0219	0.0529	0.846
VLPL	0.0284	0.0636	0.981
VLAB	0.0286	0.0636	0.896
VLPN	0.0287	0.0636	0.896
V3TG	0.0429	0.0934	0.815
V1FC	0.0447	0.0955	1.013
V2TG	0.0524	0.1058	0.908
VLTG	0.0567	0.1118	0.886
V1CH	0.0584	0.1118	0.982
VLCH	0.0606	0.1139	0.831
VLFC	0.0687	0.1270	0.824
V2CH	0.0872	0.1586	0.784

In the screening for gender-separated sub-cohort changes, we were able to locate the initially identified features of the full cohort, namely H4FC, H4CH, and L2CH (Table A2). Hereby, we may observe that the discovered features have fallen into gender dependency as following – HDL-4 parameters are predominately higher in MCI-female patients (Figure 4), while L2CH parameter showed the highest significance in the context of the male participants of the study. Overall, the observed changes for the female portion of patients is more enriched with significant (by raw *p* value) findings than ones in males. This finding goes in line with generally known features of AD-driven dementia processes that affect the patients asymmetrically when the gender factor is in the consideration [56].

Other changes observed are caused by alterations in HDL fraction of lipoprotein variables that were higher in female MCI subjects (Table A2), while in the males’ sub-cohort we saw LDL fraction of lipoproteins to be elevated in the AD patients.

**Table A2:** ANOVA series (*p*_ANOVA_ < 0.01) highlights variables based on the male-female analysis within three-group comparisons (AD, Con, and MCI) for the dataset from the whole cohort lipoprotein data. Data entries from the Supplementary Materials Table S3 are highlighted in bold.

Variable, Female	*p* Value, Female	*p* (FDR Adjusted), Female	VIP (PLS-DA), Female
H3FC	0.0027	0.1947	0.227
H2FC	0.0035	0.1947	0.075
HDA1	0.0093	0.2594	0.074
TPA1	0.0110	0.2594	0.001
HDPL	0.0139	0.2594	0.278
HDCH	0.0145	0.2594	0.188
**H4FC**	**0.0164**	**0.2594**	**0.112**
H2A1	0.0212	0.2715	0.331
V1FC	0.0228	0.2715	0.486
H3CH	0.0245	0.2715	0.097
L1FC	0.0275	0.2770	0.817
V1PL	0.0309	0.2855	1.385
V1TG	0.0365	0.2932	0.012
TPA2	0.0370	0.2932	0.098
H2A2	0.0452	0.3307	0.174
HDA2	0.0477	0.3307	0.093
H3PL	0.0507	0.3307	0.192
H1A2	0.0550	0.3390	0.449
**H4CH**	**0.0677**	**0.3666**	**0.089**
H3A1	0.0709	0.3666	0.046
V2FC	0.0729	0.3666	3.183
HDFC	0.0746	0.3666	0.071
L3FC	0.0760	0.3666	1.114
VLTG	0.0844	0.3770	0.414
V1CH	0.0895	0.3770	1.113
H1FC	0.0906	0.3770	0.119
V2TG	0.0917	0.3770	0.235
V2PL	0.0956	0.3790	0.932
**Variable, Male**	***p* Value, Male**	***p* (FDR Adjusted), Male**	**VIP (PLS-DA), Male**
**L2CH**	**0.0046**	**0.2436**	**2.759**
L2AB	0.0066	0.2436	2.274
L2PN	0.0066	0.2436	2.275
L2PL	0.0119	0.3304	2.151
L3PN	0.0205	0.3664	2.848
L3CH	0.0240	0.3664	3.185
L2FC	0.0257	0.3664	2.255
L3AB	0.0264	0.3664	2.772
L3PL	0.0384	0.4734	2.410
V5CH	0.0539	0.5987	2.935
L3FC	0.0646	0.6520	1.921
V5TG	0.0748	0.6922	1.189
H4TG	0.0915	0.7814	1.216

Therefore, we conclude that AD can be stronger characterized by elevations in female patients of HDL cholesterol, HDL-1 and HDL-2 subfraction parameters, HDL Apo-A1 apolipoprotein, and HDL phospholipid concentrations.

At the same time, our main findings (Figure 4) of dementia-driven changes within the studied cohort were connected with other descriptors such as HDL-4 and triglycerides levels which in fact were not significantly altered by the factor of patients’ gender.
