# Supplementary material for: Quantitative NMR-Based Lipoprotein Analysis Identifies Elevated HDL-4 and Triglycerides in the Serum of Alzheimer’s Disease Patients
Source: Int J Mol Sci. 2022 Oct 18;23(20):12472. doi: 10.3390/ijms232012472 (PMC9604278; doi:10.3390/ijms232012472)
Supplement: Supplementary file 1 [file ijms-23-12472-s001.zip › 3-gr_PCA_sPLS-DA_ANOVA_Figure_S1.pdf]

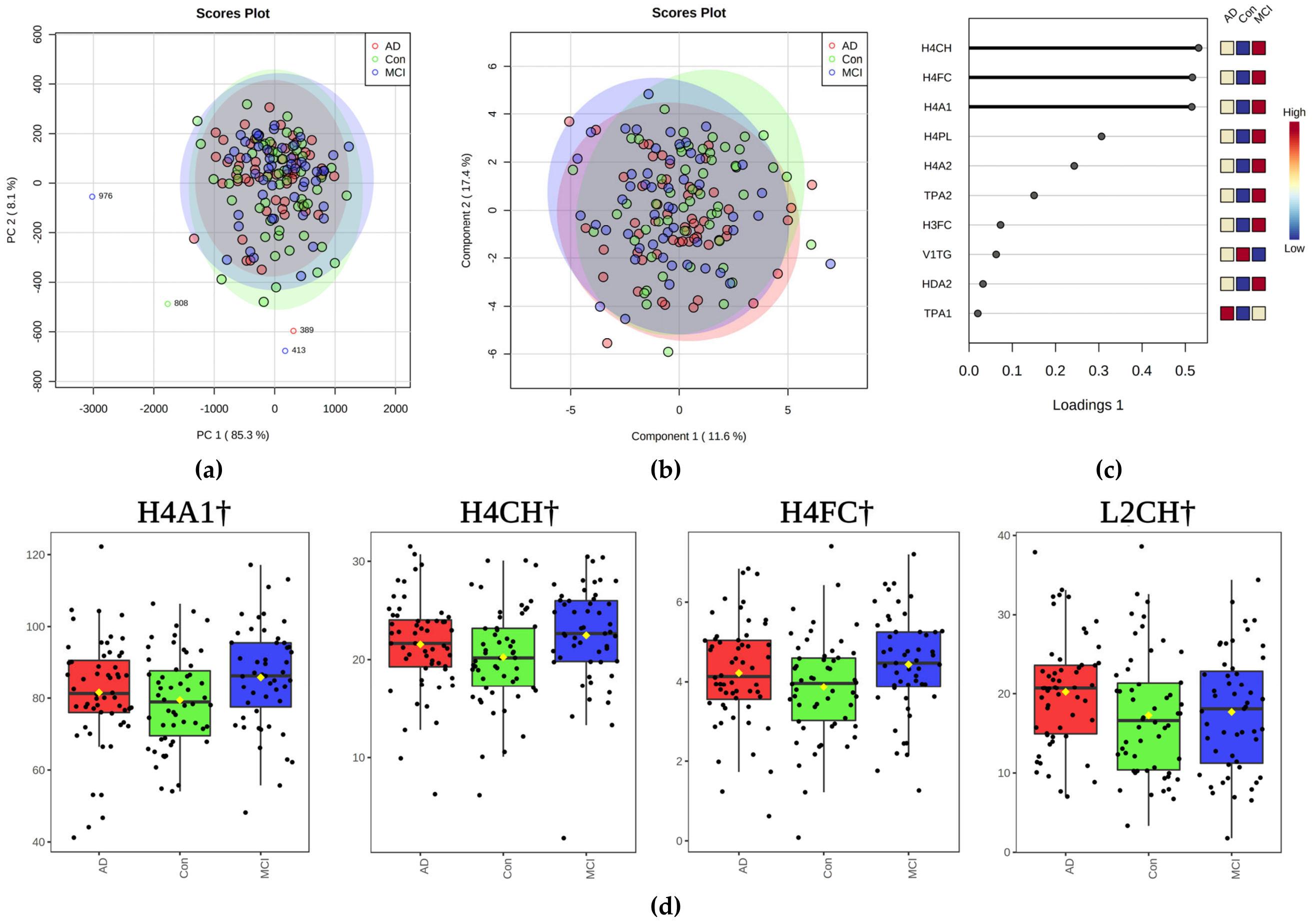

Figure S1. Multivariate analysis of three-group comparison of lipoprotein data analysis plotted via principle component analysis (PCA) scores plot (Panel a, outliers shown), via the sparse partial least-squares discrimination analysis (sPLS-DA) regression model analysis scores plot (Panel b), and loadings plot (Panel c). The ANOVA box plots of statistically significant parameters are shown (Panel d). The significances are taken from the Supplementary Materials Table S3. p values: † $p < 0.10$.
