# Supplementary material for: Quantitative NMR-Based Lipoprotein Analysis Identifies Elevated HDL-4 and Triglycerides in the Serum of Alzheimer’s Disease Patients
Source: Int J Mol Sci. 2022 Oct 18;23(20):12472. doi: 10.3390/ijms232012472 (PMC9604278; doi:10.3390/ijms232012472)
Supplement: Supplementary file 1 [file ijms-23-12472-s001.zip › 3-gr_whole_cohort_top25_Spearman_corrs_MMSE_Figure_S3.pdf]

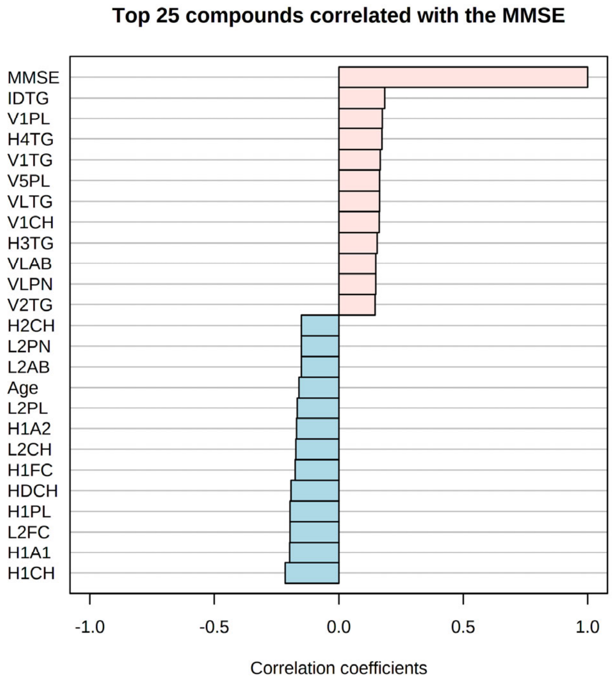

Figure S3. Three-group full cohort dataset comparison of top 25 Spearman correlations performed in the context of MMSE score levels and visualized via a PatternSearch plot.
