# Supplementary material for: Quantitative NMR-Based Lipoprotein Analysis Identifies Elevated HDL-4 and Triglycerides in the Serum of Alzheimer’s Disease Patients
Source: Int J Mol Sci. 2022 Oct 18;23(20):12472. doi: 10.3390/ijms232012472 (PMC9604278; doi:10.3390/ijms232012472)
Supplement: Supplementary file 1 [file ijms-23-12472-s001.zip › AD-MCI_with_CSF_biomarker_data_Table_S5.pdf]

Table S5. Volcano analysis (FC > 1.20) variables based on the AD-MCI sub-cohort analysis with included CSF biomarker data and clinical metadata.

Variable	p value	p (FDR adjusted) value	VIP (oPLS-DA) scores
MMSE***	$5.70 \cdot 10^{-5}$	0.0066148	2.50638
CSF h-Tau***	$0.92 \cdot 10^{-3}$	0.053632	3.33104
CSF p-Tau**	0.0015	0.056357	3.18209
CSFAβ1-42**	0.0038	0.11092	2.04571
V1CH*	0.0313	0.61622	1.71706
V1TG*	0.0373	0.61622	1.69412
VLPL†	0.0517	0.61622	1.62185
V1PL†	0.0524	0.61622	1.53238
VLFC†	0.0565	0.61622	1.52974
V2FC†	0.0640	0.61622	1.25871
VLTG†	0.0701	0.61622	1.43322
IDTG†	0.0781	0.61622	1.36563
IDPL†	0.0852	0.61622	1.51304
V2PL†	0.0866	0.61622	1.34341
V1FC†	0.0869	0.61622	1.45258
V2TG†	0.0878	0.61622	1.33780
V3FC†	0.0903	0.61622	1.43120

p values: *** p < 0.0001; * p < 0.05; † p < 0.10.
