# Supplementary material for: Quantitative NMR-Based Lipoprotein Analysis Identifies Elevated HDL-4 and Triglycerides in the Serum of Alzheimer’s Disease Patients
Source: Int J Mol Sci. 2022 Oct 18;23(20):12472. doi: 10.3390/ijms232012472 (PMC9604278; doi:10.3390/ijms232012472)
Supplement: Supplementary file 1 [file ijms-23-12472-s001.zip › ANOVA-significant_parameters_full_cohort_MMSE_age_Table_S4.pdf]

Table S4. ANOVA-significant parameters based on the full cohort data, MMSE, and age values.

Variable	p value	FDR value	VIP (PLS-DA) scores
MMSE***	$2.76 \cdot 10^{-20}$	$3.12 \cdot 10^{-18}$	4.0043
L3CH*	0.046	0.70051	2.7724
L2CH*	0.049	0.70051	2.4750
L3PN †	0.058	0.70051	2.4315
L3PL †	0.063	0.70051	2.0424
L3AB †	0.067	0.70051	2.3621
L3FC †	0.078	0.70051	1.6632
V2FC †	0.083	0.70051	2.1790
V1TG †	0.085	0.70051	0.8609
IDTG †	0.089	0.70051	0.3984
H4A1 †	0.094	0.70051	0.7199

p values: *** $p < 0.0001$; * $p < 0.05$; † $p < 0.10$.
