# Supplementary material for: Quantitative NMR-Based Lipoprotein Analysis Identifies Elevated HDL-4 and Triglycerides in the Serum of Alzheimer’s Disease Patients
Source: Int J Mol Sci. 2022 Oct 18;23(20):12472. doi: 10.3390/ijms232012472 (PMC9604278; doi:10.3390/ijms232012472)
Supplement: Supplementary file 1 [file ijms-23-12472-s001.zip › ANOVA-significant_parameters_without_log_normalization_Table_S3.pdf]

**Table S3.** ANOVA-significant parameters based on the full cohort data without logarithmic normalization.

| Variable | p value | FDR value | VIP (PLS-DA) scores |
| --- | --- | --- | --- |
| H4A1 † | 0.064867 | 0.95172 | 0.5019 |
| H4CH † | 0.072953 | 0.95172 | 0.1032 |
| H4FC † | 0.07529 | 0.95172 | 0.0241 |
| L2CH † | 0.080319 | 0.95172 | 0.0690 |

p values: † $p < 0.10$.
