# Supplementary material for: Quantitative NMR-Based Lipoprotein Analysis Identifies Elevated HDL-4 and Triglycerides in the Serum of Alzheimer’s Disease Patients
Source: Int J Mol Sci. 2022 Oct 18;23(20):12472. doi: 10.3390/ijms232012472 (PMC9604278; doi:10.3390/ijms232012472)
Supplement: Supplementary file 1 [file ijms-23-12472-s001.zip › IVDr_lipoprotein_acronyms_Table_S1.pdf]

Table S1. Annotation of the abbreviations used by the IVDr lipoprotein profiling analysis.

Nr.	Name	Extended name	Unit
1	ABA1	Apolipoprotein-B100/Apolipoprotein-A1	-
2	H1A1	Apolipoprotein-A1 HDL-1	mg/dL
3	H1A2	Apolipoprotein-A2 HDL-1	mg/dL
4	H1CH	Cholesterol HDL-1	mg/dL
5	H1FC	Free Cholesterol HDL-1	mg/dL
6	H1PL	Phospholipids HDL-1	mg/dL
7	H1TG	Triglycerides HDL-1	mg/dL
8	H2A1	Apolipoprotein-A1 HDL-2	mg/dL
9	H2A2	Apolipoprotein-A2 HDL-2	mg/dL
10	H2CH	Cholesterol HDL-2	mg/dL
11	H2FC	Free Cholesterol HDL-2	mg/dL
12	H2PL	Phospholipids HDL-2	mg/dL
13	H2TG	Triglycerides HDL-2	mg/dL
14	H3A1	Apolipoprotein-A1 HDL-3	mg/dL
15	H3A2	Apolipoprotein-A2 HDL-3	mg/dL
16	H3CH	Cholesterol HDL-3	mg/dL
17	H3FC	Free Cholesterol HDL-3	mg/dL
18	H3PL	Phospholipids HDL-3	mg/dL
19	H3TG	Triglycerides HDL-3	mg/dL
20	H4A1	Apolipoprotein-A1 HDL-4	mg/dL
21	H4A2	Apolipoprotein-A2 HDL-4	mg/dL
22	H4CH	Cholesterol HDL-4	mg/dL
23	H4FC	Free Cholesterol HDL-4	mg/dL
24	H4PL	Phospholipids HDL-4	mg/dL
25	H4TG	Triglycerides HDL-4	mg/dL
26	HDA1	HDL-Apolipoprotein-A1	mg/dL
27	HDA2	HDL-Apolipoprotein-A2	mg/dL
28	HDCH	HDL-Cholesterol	mg/dL
29	HDFC	HDL Free Cholesterol	mg/dL
30	HDPL	HDL Phospholipids	mg/dL
31	HDTG	HDL Triglycerides	mg/dL
32	IDAB	IDL-Apolipoprotein-B100	mg/dL
33	IDCH	IDL Cholesterol	mg/dL
34	IDFC	IDL Free Cholesterol	mg/dL
35	IDPL	IDL Phospholipids	mg/dL
36	IDPN	ILDL Particle Number	nmol/L
37	IDTG	IDL Triglycerides	mg/dL
38	L1AB	Apolipoprotein-B100 LDL-1	mg/dL
39	L1CH	Cholesterol LDL-1	mg/dL
40	L1FC	Free Cholesterol LDL-1	mg/dL
41	L1PL	Phospholipids LDL-1	mg/dL
42	L1PN	Particle Number LDL-1	nmol/L

43	L1TG	Triglycerides LDL-1	mg/dL
44	L2AB	Apolipoprotein-B100 LDL-2	mg/dL
45	L2CH	Cholesterol LDL-2	mg/dL
46	L2FC	Free Cholesterol LDL-2	mg/dL
47	L2PL	Phospholipids LDL-2	mg/dL
48	L2PN	Particle Number LDL-2	nmol/L
49	L2TG	Triglycerides LDL-2	mg/dL
50	L3AB	Apolipoprotein-B100 LDL-3	mg/dL
51	L3CH	Cholesterol LDL-3	mg/dL
52	L3FC	Free Cholesterol LDL-3	mg/dL
53	L3PL	Phospholipids LDL-3	mg/dL
54	L3PN	Particle Number LDL-3	nmol/L
55	L3TG	Triglycerides LDL-3	mg/dL
56	L4AB	Apolipoprotein-B100 LDL-4	mg/dL
57	L4CH	Cholesterol LDL-4	mg/dL
58	L4FC	Free Cholesterol LDL-4	mg/dL
59	L4PL	Phospholipids LDL-4	mg/dL
60	L4PN	Particle Number LDL-4	nmol/L
61	L4TG	Triglycerides LDL-4	mg/dL
62	L5AB	Apolipoprotein-B100 LDL-5	mg/dL
63	L5CH	Cholesterol LDL-5	mg/dL
64	L5FC	Free Cholesterol LDL-5	mg/dL
65	L5PL	Phospholipids LDL-5	mg/dL
66	L5PN	Particle Number LDL-5	nmol/L
67	L5TG	Triglycerides LDL-5	mg/dL
68	L6AB	Apolipoprotein-B100 LDL-6	mg/dL
69	L6CH	Cholesterol LDL-6	mg/dL
70	L6FC	Free Cholesterol LDL-6	mg/dL
71	L6PL	Phospholipids LDL-6	mg/dL
72	L6PN	Particle Number LDL-6	nmol/L
73	L6TG	Triglycerides LDL-6	mg/dL
74	LDAB	LDL-Apolipoprotein-B100	mg/dL
75	LDCH	LDL-Cholesterol	mg/dL
76	LDFC	LDL Free Cholesterol	mg/dL
77	LDHD	LDL-cholesterol/HDL-cholesterol	-
78	LDPL	LDL Phospholipids	mg/dL
79	LDPN	LDL Particle Number	nmol/L
80	LDTG	LDL Triglycerides	mg/dL
81	TBPN	Total Particle Number (apolipoprotein-B100 carrying particles)	nmol/L
82	TPA1	Total Plasma Apolipoprotein-A1	mg/dL
83	TPA2	Total Plasma Apolipoprotein-A2	mg/dL
84	TPAB	Total Plasma Apolipoprotein-B100	mg/dL
85	TPCH	Total Plasma Cholesterol	mg/dL
86	TPTG	Total Plasma Triglycerides	mg/dL
87	V1CH	Cholesterol VLDL-1	mg/dL

88	V1FC	Free Cholesterol VLDL-1	mg/dL
89	V1PL	Phospholipids VLDL-1	mg/dL
90	V1TG	Triglycerides VLDL-1	mg/dL
91	V2CH	Cholesterol VLDL-2	mg/dL
92	V2FC	Free Cholesterol VLDL-2	mg/dL
93	V2PL	Phospholipids VLDL-2	mg/dL
94	V2TG	Triglycerides VLDL-2	mg/dL
95	V3CH	Cholesterol VLDL-3	mg/dL
96	V3FC	Free Cholesterol VLDL-3	mg/dL
97	V3PL	Phospholipids VLDL-3	mg/dL
98	V3TG	Triglycerides VLDL-3	mg/dL
99	V4CH	Cholesterol VLDL-4	mg/dL
100	V4FC	Free Cholesterol VLDL-4	mg/dL
101	V4PL	Phospholipids VLDL-4	mg/dL
102	V4TG	Triglycerides VLDL-4	mg/dL
103	V5CH	Cholesterol VLDL-5	mg/dL
104	V5FC	Free Cholesterol VLDL-5	mg/dL
105	V5PL	Phospholipids VLDL-5	mg/dL
106	V5TG	Triglycerides VLDL-5	mg/dL
107	VLAB	VLDL-Apolipoprotein-B100	mg/dL
108	VLCH	VLDL Cholesterol	mg/dL
109	VLFC	VLDL Free Cholesterol	mg/dL
110	VLPL	VLDL Phospholipids	mg/dL
111	VLPN	VLDL Particle Number	nmol/L
112	VLTG	VLDL Triglycerides	mg/dL
