# Supplementary material for: Quantitative NMR-Based Lipoprotein Analysis Identifies Elevated HDL-4 and Triglycerides in the Serum of Alzheimer’s Disease Patients
Source: Int J Mol Sci. 2022 Oct 18;23(20):12472. doi: 10.3390/ijms232012472 (PMC9604278; doi:10.3390/ijms232012472)
Supplement: Supplementary file 1 [file ijms-23-12472-s001.zip › top25_PLS-DA_log_oPLS-DA_AD-MCI_Figure_S2.pdf]

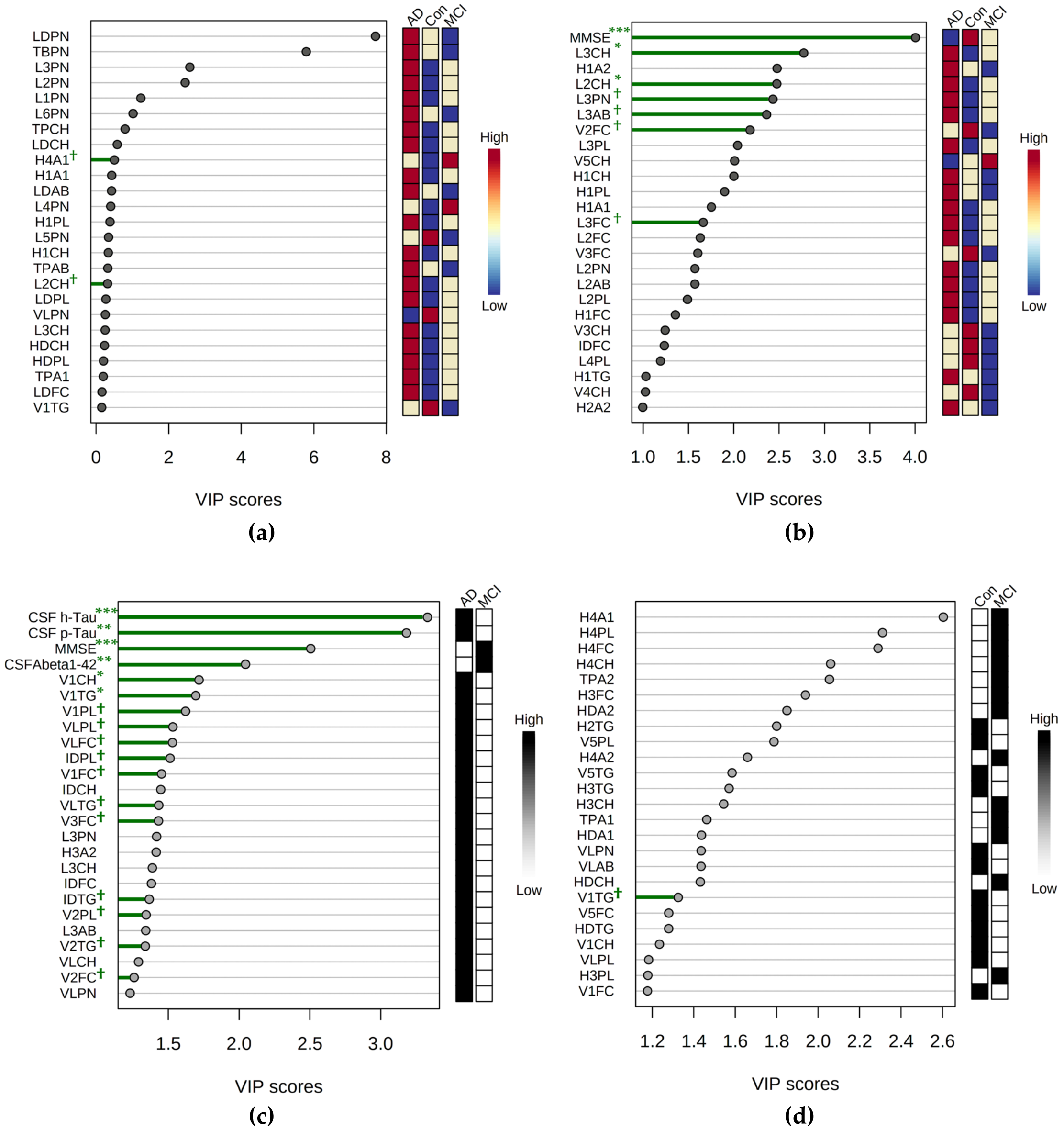

Figure S2. Four comparisons of top 25 lipoprotein parameters from the results of regression model analysis via PLS-DA (in color, Panel a – whole cohort direct comparison of the acquired data, Panel b – whole cohort comparison of logarithmically normalized data) and oPLS-DA (Panel c – AD-MCI patient group comparison, Panel d – MCI and controls groups comparison) applied to find lipoproteins distinguishing patient groups.
