# Supplementary material for: Quantitative NMR-Based Lipoprotein Analysis Identifies Elevated HDL-4 and Triglycerides in the Serum of Alzheimer’s Disease Patients
Source: Int J Mol Sci. 2022 Oct 18;23(20):12472. doi: 10.3390/ijms232012472 (PMC9604278; doi:10.3390/ijms232012472)
Supplement: Supplementary file 1 [file ijms-23-12472-s001.zip › Volcano-significant_parameters_MCI-Con_full_cohort_Table_S7.pdf]

**Table S7.** Volcano-significant parameters (MCI-Con) based on the full cohort data.

| Variable | p value | p (FDR adjusted) value | VIP (oPLS-DA) scores |
|---|---|---|---|
| V1TG* | 0.0377 | 0.72537 | 1.32497 |
| IDTG† | 0.0633 | 0.72537 | 1.08419 |

p values: * $p < 0.05$; † $p < 0.10$.
